# Supplementary material for: Distinct stages of synapse elimination are induced by burst firing of CA1 neurons and differentially require MEF2A/D
Source: eLife. 2017 Sep 13;6:e26278. doi: 10.7554/eLife.26278 (PMC5608508; doi:10.7554/eLife.26278)
Supplement: Supplementary file 1. [file elife-26278-supp1.docx]

**Supplementary File 1, Related to Figure 1. Raw electrophysiological measurements in untransfected (U) or transfected (T) hippocampal CA1 neurons**

|  |  | **Evoked**  **EPSC**  **(pA)** | **PPF**  **(S2/S1)** | **mEPSC**  **Amp**  **(pA)** | **mEPSC**  **Freq**  **(Hz)** | **R_n_**  **(MΩ)** | **I_LED_**  **(pA)** |
| --- | --- | --- | --- | --- | --- | --- | --- |
|  |  |  |  |  |  |  |  |
| ***No PPS*** |  |  |  |  |  |  |  |
| WT | U | 42±9  (12) | 1.2±0.1  (12) | 12±0.6  (19) | 2.0±0.5  (19) | 520±63  19) | n.d. |
|  | T | 40±4 | 1.2±0.1 | 13±0.8 | 1.9±0.4 | 409±46** |  |
| *Mef2a/d^fl/fl^* | U | 55±5  (17) | 1.5 ±0.1  (14) | 12±0.6  (17) | 2.8±0.6  (17) | 417±29  (17) | n.d. |
|  | T | 56±8 | 1.2±0.1* | 12±0.9 | 2.2±0.4 | 342±30 |  |
| ***1 hr PPS*** |  |  |  |  |  |  |  |
| WT | U | 88±15 (15) | 1.5±0.1  (14) | 15±1.3  (13) | 2.7±0.6  (13) | 160±23  (15) | 1300±173  (15) |
|  | T | 40±8* | 1.2±0.1 | 13±1.0 | 1.5±0.3* | 192±32 |  |
| *Mef2a/d^fl/fl^* | U | 76±11  (16) | 1.4 ±0.1  (13) | 12±1.2  (16) | 1.5±0.3  (16) | 167±21  (16) | 1060±128  (16) |
|  | T | 71±13 | 1.3±0.1 | 12±0.9 | 1.6±0.3 | 148±20 |  |
| ***6 hr PPS*** |  |  |  |  |  |  |  |
| WT | U | 61±8  (18) | 1.4±0.1  (17) | 11±0.7  (18) | 0.93±0.1  (18) | 352±33  (18) | 705±93  (15) |
|  | T | 40±5* | 1.4±0.1 | 11±0.5 | 0.61±0.1** | 243±23* |  |
| *Mef2a/d^fl/fl^* | U | 76±11  (20) | 1.4 ±0.1  (19) | 15±1.2  (19) | 0.86±0.2  (19) | 163±15  (19) | 599±68  (19) |
|  | T | 48±10 | 1.3±0.1 | 14±1.6 | 0.93±0.2 | 196±16 |  |
| ***24 hr PPS*** |  |  |  |  |  |  |  |
| WT | U | 82±17 (17) | 1.4±0.1  (16) | 14±1.1  (19) | 1.1±0.3  (19) | 209±14  (17) | 557±160  (5) |
|  | T | 38±6** | 1.4±0.1 | 13±1.1 | 0.73±0.2* | 224±29 |  |
| *Mef2a/d^fl/fl^* | U | 76±11  (24) | 1.5 ±0.1  (22) | 14±0.8  (20) | 1.3±0.3  (20) | 246±28  (24) | 732±127  (17) |
|  | T | 48±12* | 1.3±0.1 | 12±0.8 | 0.81±0.2* | 240±28 |  |

^#^not corrected for junction potential; data are presented as mean±SEM; *p<0.05, **p<0.01, ***p<0.001, paired t-test or Wilcoxon matched-pairs signed rank test depending on Gaussian distribution; number of cell pairs indicated in parentheses. I_LED_ is the inward current evoked by a blue light pulse as described in methods (200 msec).

**Table S2, Related to Figure 2. Raw electrophysiological measurements in untransfected (U) or transfected (T) hippocampal CA1 neurons**

|  |  | **AMPA**  **eEPSC**  **(pA)** | **NMDA**  **eEPSC**  **(pA)** | **R_n_**  **(MΩ)** | **I_LED_**  **(pA)** |
| --- | --- | --- | --- | --- | --- |
| ***1 hr PPS*** | | | | | |
| ***3-7 hr after PPS onset*** | | | | | |
| WT | U | 92±12  (22) | 122±14  (22) | 233±24  (22) | 367±75  (22) |
|  | T | 60±7* | 112±16 | 223±22 |  |
| *Mef2a/d^fl/fl^* | U | 93±13  (19) | 111±17  (20) | 244±33  (20) | 612±121  (19) |
|  | T | 64±9* | 96±13 | 215±22 |  |
| ***12-16 hr after PPS onset*** | | | | | |
| WT | U | 85±9 (13) | 110±34  (13) | 204±30  (13) | 1179±216  (10) |
|  | T | 41±7** | 71±18 | 203±33 |  |
| ***24 hr after PPS onset*** | | | | | |
| WT | U | 138±24  (13) | 139±30  (13) | 169±19  (13) | 1408±238  (12) |
|  | T | 52±8** | 110±23 | 187±16 |  |
| ***24hr PPS*** | | | | | |
| ***24hr after PPS onset*** | | | | | |
| WT | U | 73±9 (21) | 56±8  (14) | 225±26  (14) | 716±189  (14) |
|  | T | 42±7* | 33±4** | 292±22** |  |
| ***48hr after PPS onset*** | | | | | |
| WT | U | 58±8 (22) | 115±14  (20) | 345±29  (25) | 350±74  (21) |
|  | T | 35±4** | 71±9** | 334±30 |  |
| *Mef2a/d^fl/fl^* | U | 81±14  (16) | 169±23  (16) | 232±31  (16) | 474±116  (16) |
|  | T | 32±6** | 94±22* | 228±25 |  |

^#^not corrected for junction potential; data are presented as mean±SEM; *p<0.05, **p<0.01, paired t-test or Wilcoxon matched-pairs signed rank test depending on Gaussian distribution; number of cell pairs indicated in parentheses. I_LED_ is the inward current evoked by a blue light pulse as described in methods (200 msec).

**Table S3, Related to Figure 5 and Figure S5. Raw electrophysiological measurements in untransfected (U) or transfected (T) hippocampal CA1 neurons**

|  |  | **Evoked**  **EPSC**  **(pA)** | **PPF**  **(S2/S1)** | **mEPSC**  **Amp**  **(pA)** | **mEPSC**  **Freq**  **(Hz)** | **R_n_**  **(MΩ)** | **I_LED_**  **(pA)** |
| --- | --- | --- | --- | --- | --- | --- | --- |
| **Wildtype** |  |  |  |  |  |  |  |
| ***1hr PPS-Nifedipine*** |  |  |  |  |  |  |  |
| Vehicle (0.1%DMSO) | U | 49±8  (12) | 1.2±0.2  (7) | 15±0.7  (16) | 1.4±0.5  (16) | 183±13  (21) | 997±122  (21) |
|  | T | 29±8* | 1.3±0.2 | 12±0.4*** | 0.73±0.2*** | 171±11 |  |
| 20μM Nifedipine | U | 53±8  (14) | 1.1 ±0.2  (12) | 14±0.6  (19) | 1.1±0.2  (19) | 178±8  (19) | 910±46  (23) |
|  | T | 44±9 | 0.99±0.1 | 12±0.4* | 0.98±0.2 | 184±11 |  |
| ***1hr PPS-DRB*** |  |  |  |  |  |  |  |
| Vehicle (0.2%DMSO) | U | ### | ### | 13±0.6  (18) | 1.2±0.3  (18) | 247±18  (23) | 676±108  (22) |
|  | T | ### | ### | 12±0.7* | 0.84±0.2* | 217±16 |  |
| 160μM DRB | U | ### | ### | 13±0.6  (14) | 0.64±0.1  (14) | 224±20  (21) | 653±79  (21) |
|  | T | ### | ### | 12±0.5 | 0.54±0.1 | 214±14 |  |
| ***1hr PPS-ActD*** |  |  |  |  |  |  |  |
| Vehicle (0.01%DMSO) | U | ### | ### | 14±0.5  (16) | 1.7±0.3  (16) | 181±14  (20) | 884±121  (20) |
|  | T | ### | ### | 12±0.4 | 0.93±0.2**** | 201±21 |  |
| 1μM ActD | U | ### | ### | 14±0.8  (16) | 1.2±0.3  (16) | 159±11  (20) | 713±118  (20) |
|  | T | ### | ### | 13±0.8 | 1.0±0.3 | 178±15 |  |
| ***24hr PPS-Aniso*** |  |  |  |  |  |  |  |
| Vehicle  (0.1%DMSO) | U | 61±5  (14) | 1.7±0.2  (11) | 14±0.8  (16) | 0.69±0.2  (16) | 284±23  (20) | 509±63  (18) |
|  | T | 38±8* | 1.7±0.2 | 14±0.7 | 0.52±0.2* | 268±16 |  |
| 20μM Anisomycin | U | 61±7  (14) | 1.5 ±0.1  (10) | 14±0.7  (14) | 0.91±0.2  (14) | 226±13  (16) | 470±38  (16) |
|  | T | 51±12 | 1.6±0.1 | 14±0.8 | 0.92±0.2 | 242±14 |  |

^#^not corrected for junction potential; data are presented as mean±SEM; *p<0.05, **p<0.01, ***p<0.001, ****p<0.0001, paired t-test or Wilcoxon matched-pairs signed rank test depending on Gaussian distribution; number of cell pairs indicated in parentheses. #### not reported; see text. I_LED_ is the inward current evoked by a blue light pulse as described in methods (200 msec).

**Table S4, Related to Figure 6 and Figure S6. Raw electrophysiological measurements in untransfected (U) or transfected (T) hippocampal CA1 neurons**

|  |  | **Evoked**  **EPSC**  **(pA)** | **PPF**  **(S2/S1)** | **mEPSC**  **Amp**  **(pA)** | **mEPSC**  **Freq**  **(Hz)** | **R_n_**  **(MΩ)** | **I_LED_**  **(pA)** |
| --- | --- | --- | --- | --- | --- | --- | --- |
| ***1hr PPS*** |  |  |  |  |  |  |  |
| WT | U | 50±5  (17) | 1.3±0.1  (15) | 15±0.6  (16) | 1.3±0.3  (16) | 174±10  (25) | 789±110  (21) |
|  | T | 31±5** | 1.5±0.1 | 14±0.6 | 0.76±0.2** | 187±10 |  |
| *Arc* KO | U | 38±5  (16) | 1.5 ±0.2  (11) | 15±0.6  (15) | 1.2±0.3  (15) | 181±11  (24) | 748±119  (19) |
|  | T | 33±9 | 1.6±0.2 | 14±0.7 | 0.98±0.2 | 184±15 |  |
| ***0hr PPS*** |  |  |  |  |  |  |  |
| *Arc* KO+Arc cDNA | U | 64±7  (14) | 1.5 ±0.1  (9) | 15±0.8  (14) | 1.3±0.3  (14) | 214±17  (21) | 499±71  (21) |
|  | T | 54±8 | 1.5±0.2 | 15±0.9 | 1.1±0.3 | 192±17 |  |
| *Mef2a/d^fl/fl^*+Arc cDNA | U | ### | ### | 17±1.6  (9) | 1.7±0.3  (9) | 148±23  (9) | 816±150  (12) |
|  | T | ### | ### | 14±1.8 | 1.5±0.5 | 196±22 |  |
| ***1hr PPS*** |  |  |  |  |  |  |  |
| *Arc* KO+Arc cDNA | U | 74±10  (14) | 1.7 ±0.1  (8) | 16±0.8  (15) | 1.7±0.4  (15) | 256±24  (15) | 678±79  (18) |
|  | T | 43±8* | 1.7±0.2 | 15±0.6 | 0.87±0.2** | 245±31 |  |
| *Mef2a/d^fl/fl^*+Arc cDNA | U | ### | ### | 18±2  (13) | 1.6±0.4 (13) | 141±15  (13) | 935±291  (9) |
|  | T | ### | ### | 19±2 | 1.7±0.5 | 177±16* |  |
| ***24hr PPS*** |  |  |  |  |  |  |  |
| WT | U | 57±7  (23) | 1.2 ±0.1  (13) | 16±1  (18) | 1.9±0.5  (17) | 194±10  (34) | 674±82  (33) |
|  | T | 22±10**** | 1.2±0.2 | 14±0.8 | 1.1±0.4* | 170±12* |  |
| *Arc* KO | U | 51±6  (24) | 1.4±0.1  (15) | 15±0.6  (22) | 0.99±0.2  (22) | 183±9  (39) | 586±53  (37) |
|  | T | 34±7** | 1.4±0.2 | 14±0.7 | 0.97±0.2 | 193±8 |  |

^#^not corrected for junction potential; data are presented as mean±SEM; *p<0.05, **p<0.01, ***p<0.001, ****p<0.0001, paired t-test or Wilcoxon matched-pairs signed rank test depending on Gaussian distribution; number of cell pairs indicated in parentheses. #### not reported; see text. I_LED_ is the inward current evoked by a blue light pulse as described in methods (200 msec).

**References:**

Elmer BM, Estes ML, Barrow SL, McAllister AK (2013) MHCI Requires MEF2 Transcription Factors to Negatively Regulate Synapse Density during Development and in Disease. J Neurosci 33:13791-13804.

Goold CP, Nicoll RA (2010) Single-cell optogenetic excitation drives homeostatic synaptic depression. Neuron 68:512-528.
